# Management of Charles Bonnet syndrome in routine eye care services

**DOI:** 10.1038/s41433-025-04215-0

**Published:** 2026-01-13

**Authors:** Lee Jones, Dominic H. ffytche, Mariya Moosajee

**Affiliations:** 1 Institute of Ophthalmology, University College London, London, UK; 2 Institute of Psychiatry, Psychology and Neuroscience, King’s College London, London, UK; 3 Moorfields Eye Hospital NHS Foundation Trust, London, UK; 4 The Francis Crick Institute, London, UK

**Keywords:** Health services, Education

Developed by UK clinicians and academics specialising in hallucination and ophthalmic health research, these guidelines integrate international evidence with real-world clinical expertise to support the identification and management of Charles Bonnet syndrome (CBS). They provide a structured framework for eye care professionals, outlining pre-emptive strategies, differential diagnostic considerations, and evidence-informed interventions to mitigate the impact of the condition. This practical, cross-disciplinary protocol is designed for routine clinical practice, promoting consistency in patient support across services.

CBS is characterised by visual hallucinations in people with visual impairment, occurring in the absence of psychiatric or cognitive disorder. These single-sensory experiences range from simple shapes or patterns to complex images of people, animals, or landscapes, with the ICD-11 definition focussing on complex experiences. CBS occurs across a wide spectrum of eye diseases associated with visual loss [1]. Current evidence suggests the underlying mechanism involves heightened excitability of the visual cortex following reduced visual input, referred to as de-afferentation or release [2]. Risk increases with greater severity of visual loss [3], although CBS may also occur in patients with relatively preserved acuity [4, 5]. Epidemiological studies estimate that up to one in five patients attending low vision services experience CBS during their sight loss journey [6].

For many patients, understanding that CBS is a recognised, non-psychiatric consequence of visual impairment provides considerable reassurance [7]. However, around one third experience distress, fear or anxiety causing disruption to daily life, a subgroup referred to as negative outcome CBS [8, 9]. These individuals face difficulties adjusting to their symptoms and are at increased risk of social withdrawal and reduced quality of life [10, 11]. Recognition and open discussions of CBS within clinical consultations are therefore essential to reduce stigma and improve patient outcomes.

**Fig. 1:**
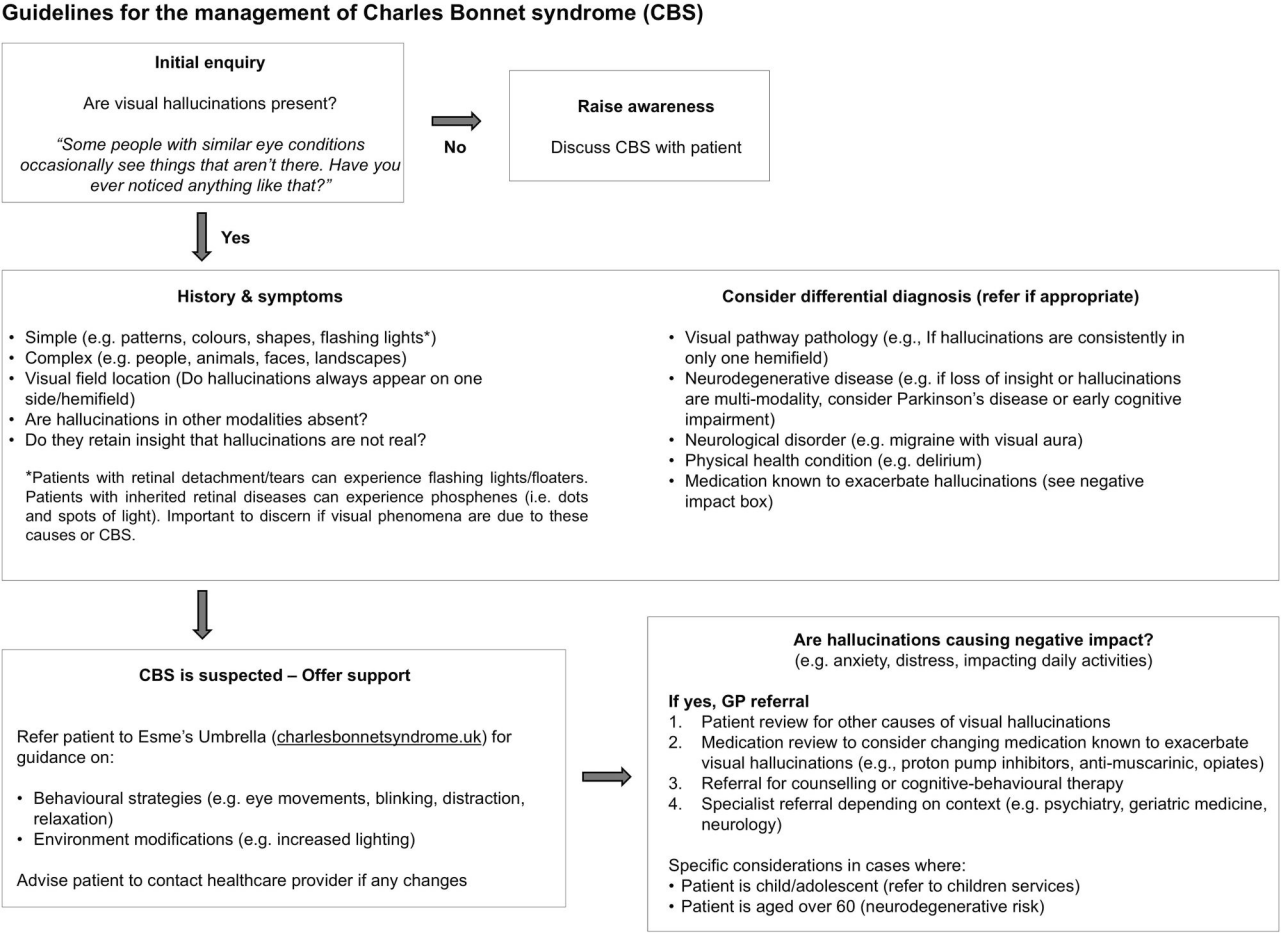
Flowchart to facilitate the identification, discussion, and management of Charles Bonnet syndrome.

Validated assessment tools such as the North-East Visual Hallucination Interview (NEVHI) [12], and the Questionnaire de Repérage du Syndrome de Charles Bonnet (QRSCB) [13], are valuable for research and detailed hallucination characterisation, but are often impractical to routinely implement in busy clinics. More importantly, there is a need for a simple, structured pathway to facilitate the identification, discussion, and management of CBS within routine ophthalmic practice, which is presented here (Fig. 1).

Most patients do not voluntarily disclose hallucinations unless directly asked, often due to concerns of an underlying psychiatric disorder. Sensitive enquiry is therefore essential. Clinicians may introduce the topic with phrasing such as *“Some people with similar eye conditions occasionally see things that aren’t really there. Have you ever noticed anything like that?”* [14]. This approach normalises the experience and encourages open discussion. Even when hallucinations are not reported, raising awareness serves a preventative role, helping patients interpret future experiences and promoting open disclosure.

When hallucinations are reported, their characteristics should be carefully explored. In CBS, hallucinations are exclusively visual and insight is retained. Presence of additional sensory modalities, delusional interpretation, or loss of insight should prompt consideration of alternative or co-existing psychiatric or cognitive conditions. A comprehensive clinical history should include onset, frequency, duration, and context, including potential triggers such as lighting conditions, fatigue, or stress [15, 16]. Assessing the emotional and functional impact is vital for monitoring progression and informing management. Findings should be documented in the medical record, including the patient’s level of insight and emotional response. Differential diagnoses must be considered. For example, flashing lights or zigzag lines (i.e., teichopsia) accompanied by transient visual changes may suggest ocular migraine, whereas a curtain-like peripheral shadow may indicate retinal detachment, an ocular emergency requiring immediate attention.

Patient education is an important intervention. Once reassured that CBS is a well-recognised and benign consequence of visual loss, ~70% of patients report minimal distress [8]. Some patients may describe hallucinations as a minor inconvenience or even as a pleasant experience [17]. Explaining CBS as spontaneous neural activity in the visual cortex compensating for reduced sensory input can enhance understanding and acceptance. For those who remain distressed, behavioural and environmental strategies may reduce symptom frequency or intensity. Anecdotal evidence suggests eye movements, blinking rapidly during an episode, or engaging in a distracting task may be effective for some people [14, 18]. Environmental modifications such as increasing ambient lighting to improve visual input may help to reduce symptoms [15]. Relaxation techniques such as mindfulness or breathing exercises may help manage anxiety [19].

If hallucinations remain distressing despite reassurance and behavioural interventions, referral for psychological support should be considered. Cognitive–behavioural therapy and other talking therapies can support coping and restore a sense of control [16]. Where hallucinations are accompanied by significant anxiety, depression, or loss of insight, referral to psychiatry or neurology is appropriate to exclude alternative or comorbid conditions.

Pharmacological interventions have shown benefit in isolated case reports, including anticonvulsants [20], antipsychotics [21], antidepressants [22], and acetylcholinesterase inhibitors [23]. Emerging evidence also indicates a potential role for neuromodulation techniques, such as transcranial direct current stimulation [24]. However, these approaches remain experimental and require further evaluation before routine clinical use can be recommended. At present, management should prioritise patient education, reassurance, behavioural strategies, and appropriate referral.

Visual hallucinations may also be medication-induced or exacerbated by certain drug classes, including proton pump inhibitors (commonly prescribed for gastrointestinal conditions) and psychotropic agents with prominent antimuscarinic effects used in neurological or psychiatric disorders. Medications should be reviewed in collaboration with the patient’s general practitioner, considering dosage adjustments or alternatives where appropriate. Drugs affecting vascular function, including nitrates, calcium channel blockers, ACE inhibitors, triptans, and sympathomimetics can alter retinal or cerebral blood flow and have been associated with shimmering, halo-like, or scintillating visual effects.

CBS is a prevalent but under-recognised condition with potential for significant psychological impact. Effective identification requires clinical vigilance, careful documentation, and exclusion of differential diagnoses. Follow-up should include reassessment of symptoms and emotional impact, with re-referral to specialist services if symptoms worsen. The accompanying flowchart provides a structured framework for recognition and management of CBS in eye care settings. This pathway aligns with consensus frameworks for visual hallucination management, including those developed by the National Institute for Health and Care Research [25]. Embedding this evidence-informed approach into routine practice can improve early recognition, reduce stigma, and promote consistent, compassionate support for individuals experiencing CBS.
